# CellularPotts.jl: simulating multiscale cellular models in Julia

**DOI:** 10.1093/bioinformatics/btad773

**Published:** 2023-12-22

**Authors:** Robert W Gregg, Panayiotis V Benos

**Affiliations:** Department of Computational and Systems Biology, University of Pittsburgh, Pittsburgh, PA 15213, United States; Department of Biomedical Informatics, University of Pittsburgh, Pittsburgh, PA 15206, United States; Department of Epidemiology, University of Florida, Gainesville, FL 32603, United States; Department of Computational and Systems Biology, University of Pittsburgh, Pittsburgh, PA 15213, United States; Department of Biomedical Informatics, University of Pittsburgh, Pittsburgh, PA 15206, United States; Department of Epidemiology, University of Florida, Gainesville, FL 32603, United States

## Abstract

**Summary:**

CellularPotts.jl is a software package written in Julia to simulate biological cellular processes such as division, adhesion, and signaling. Accurately modeling and predicting these simple processes is crucial because they facilitate more complex biological phenomena related to important disease states like tumor growth, wound healing, and infection. Here we take advantage of Cellular Potts Modeling to simulate cellular interactions and combine them with differential equations to model dynamic cell signaling patterns. These models are advantageous over other approaches because they retain spatial information about each cell while remaining computationally efficient at larger scales. Users of this package define three key inputs to create valid model definitions: a 2- or 3-dimensional space, a table describing the cells to be positioned in that space, and a list of model penalties that dictate cell behaviors. Models can then be evolved over time to collect statistics, simulated repeatedly to investigate how changing a specific property impacts cellular behavior, and visualized using any of the available plotting libraries in Julia.

**Availability and implementation:**

The CellularPotts.jl package is released under the MIT license and is available at https://github.com/RobertGregg/CellularPotts.jl. An archived version of the code (v0.3.2) at time of submission can also be found at https://doi.org/10.5281/zenodo.10407783.

## 1 Introduction

**Figure 1.**
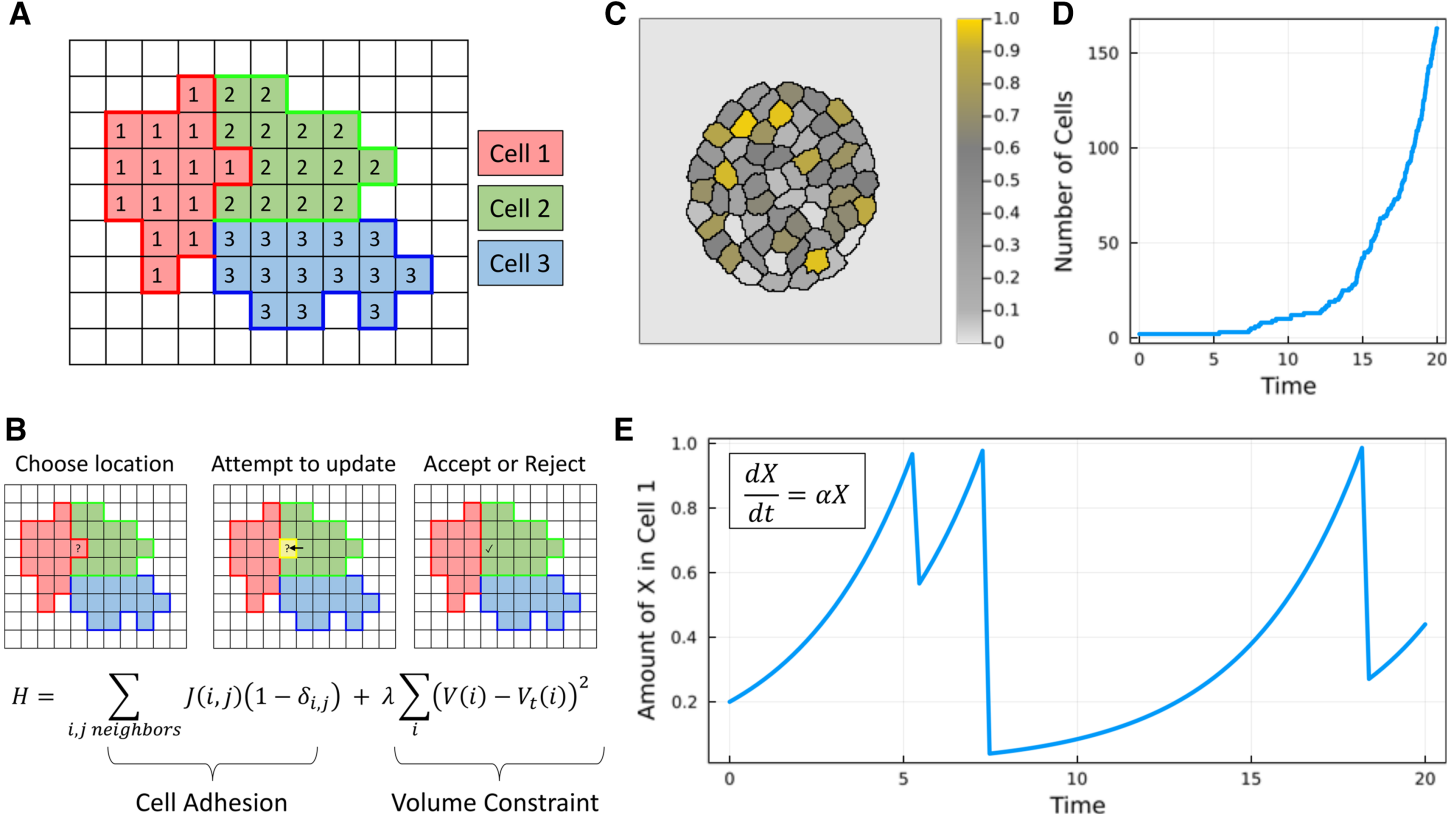
Overview of CellularPotts.jl. (A) CPMs operate on an integer grid where numbers represent cell identifiers. Here, three cells are represented on the grid. (B) The grid is updated by changing cell IDs to match neighboring IDs. Penalties are enforced on these updates (such as adhesion and volume constraints) to encourage favorable changes. The total penalty H is calculated by summing over neighbors with differing cell IDs and comparing the total cell size to a target cell size. (C–E) Give an example Cellular Potts/differential equation model which describes a growing cell population. When a theoretical protein X reaches a concentration of one, the cell divides and the protein is randomly distributed to the daughter cells. (C) Shows a snapshot of the simulation. (D) Graphs the total cell count over time. (E) Records the dynamics of the theoretical protein from one cell.

Cellular Potts Models (CPMs) are a popular method to simulate multiscale cellular behaviors because they retain some spatial information like cellular geometry but avoid the computational complexity of a full physics simulation. Their applications are general, but many researchers have focused on using these models to study cancer (e.g. angiogenesis, tumor growth, and cancer cell migration) (Szabó and Merks 2013). CPMs work by defining an integer grid where adjacent sites with the same value comprise an individual cell and locations with a value of zero represent empty regions where no cell is present (Fig. 1A). The model uses a Metropolis–Hastings algorithm to update grid sites to match their neighbors. This process depends on penalties which can, for example, encourage cells to adhere together or maintain their size (Fig. 1B). As these steps are applied to the grid, patterns observed in real cellular systems begin to emerge. The original CPM paper demonstrated how cells can sort themselves given the correct penalties (Graner and Glazier 1992). Over the next 30 years, this modeling paradigm has been updated to include behaviors like cell migration (Wortel *et al.* 2021), chemotaxis (Savill and Hogeweg 1997), and intracellular forces (Rens and Edelstein-Keshet 2019).

The goal of this package is to provide a framework to develop CPMs using a graph-based approach. As compared to other software (Morpheus (Starruß *et al.* 2014), Artistoo (Buttenschoen *et al.* 2021), CompuCell3D (Swat *et al.* 2012)), CellularPotts.jl takes a unique approach to handle common pitfalls observed when simulating these models. Specifically, our main contributions include the ability to simulate these models with arbitrary geometries, avoid cell fragmentation, and integrate with state-of-the-art differential equation libraries to facilitate the creation of multiscale models.

## 2 Methods and results

### 2.1 Main workflow

CellularPotts.jl requires three pieces of information to model a cellular system, the first being a domain that defines the space where cells exist. The default space is a rectangular grid with periodic boundary conditions, but options for a three-dimensional space or closed boundaries are available. Additionally, users can provide an image (e.g. a radiological image) to define spaces with more complex geometry.

The second requirement is a table describing what cells will be placed in the domain. Users define the names of the cells, their desired sizes (area/volume), and quantities. Positional information can be provided, otherwise cells will be placed randomly throughout the space. Custom properties like cell division rate can also be added to further inform and influence the simulation.

Finally, the user needs to specify which penalties to include in the model to encourage desired cell behaviors. In general, adhesion and volume penalties are typically added to ensure cell size and shape are maintained. Other penalties available can help maintain cell perimeter, encourage random cell migration, and move cells along concentration gradients. CellularPotts.jl is designed so that users can build their own custom penalties and expand the software’s capabilities.

With the fully defined model, users can simulate cellular behaviors by creating an animation, save the model specification for later use, or record how models evolve over time. By default, models do not save every past state because this would lead to prohibitively large save files. However, when recording is desired, CellularPotts.jl only saves how the model changes over time as opposed to a full copy of the model at each timepoint. This makes recording larger models possible at the cost of retrieving past states slower. Future directions for this package will focus on making sensitivity analysis workflows because these are difficult to perform for stochastic models.

### 2.2 Graph data structures are advantageous for CPM

CPMs are typically described using multi-dimensional arrays of integers which have several benefits including constant look-up times for a given index and contiguous memory storage to minimize cache misses. However, relying solely on one data structure for every aspect of a complex model has its disadvantages. Stepping a CPM forward in time at a specific index relies heavily on knowing information about neighboring locations, which is problematic for array data structures. A method to describe adjacent locations would be a graph data structure, which directly encodes this information through edge connections. As an example, periodic boundary conditions are solved by connecting nodes on opposite boundaries with an edge.

One critical benefit to using a graph-based approach is the identification of articulation points. CPMs are notorious for allowing cells to fragment (Durand and Guesnet 2016). This is usually addressed by lowering a modeling parameter called “temperature” which only decreases the probability of disconnections from occurring. By encoding cell space as a graph, we can simply test for articulation points (Hopcroft and Tarjan 1973) and avoid locations that would disconnect a cell. This method is guaranteed to work at any model temperature and is independent of how the user defines the geometry of the space. Additionally, for some geometries (e.g. connected and planar), we can determine articulation points independent of cell size by testing if the Euler characteristic (Wilson 1972) changes after a node is removed. This process is efficient because we only need to consider local changes to the graph (e.g. how many edges are removed).

Another benefit to using a graph-based approach can be seen with cellular division. When a cell divides, the resulting daughter cells are roughly equal in size which is difficult to simulate because cells are irregularly shaped. One method that attempts to solve this issue involves finding a line that optimally divides the cell in half. However, this method is not guaranteed to work if the cell’s boundary is concave. Conversely, by using a graph partitioning algorithm we can handle any cell shape. In general, graph partitioning is an NP hard problem, but because we are only performing a 2-way partition, polynomial time heuristic algorithms remain tractable (Karypis and Kumar 1998).

### 2.3 Easy integration with other software

CPMs operate on a discretized space and over discrete time intervals which make them difficult to combine with continuous time models like systems of ordinary differential equations (ODEs). One would typically want to couple these models together to simulate cellular and sub-cellular processes (i.e. multiscale modeling). For example, each cell could contain some theoretical protein governed by an ODE which triggers a cell division event after reaching a certain threshold (Fig. 1C–E). Most CPM software solves ODEs using Runge-Kutta or Euler methods that run in-sync with the CPM’s Monte Carlo steps. This can lead to unstable trajectories that deviate from the true solution. Julia’s DifferentialEquations.jl library (Rackauckas and Nie 2017) (and more broadly the SciML ecosystem) offers a best-in-class suite of ODE solvers that can uniquely handle discontinuous jumps and variable state systems. ODEs can evolve independently of the CPM and can even adapt if the error does not meet a specified tolerance.

Beyond solving ODEs, CellularPotts.jl can seamlessly integrate with the entire Julia library ecosystem to meet the needs of almost any user. Visualization is accomplished using the Plots.jl library, physical units could be added using the Unitful.jl library, and models could even be run in R or Python using the JuliaConnectoR or PyJulia packages, respectively. This interoperability is key to making CellularPotts.jl successful because users can customize the software to meet their specific needs.

## Data Availability

No new data were generated or analysed in support of this research.

## References

[btad773-B1] Buttenschoen A et al Artistoo, a library to build, share, and explore simulations of cells and tissues in the web browser. Elife 2021;10:e61288. 33835022 10.7554/eLife.61288 PMC8143789

[btad773-B2] Durand M , Guesnet E. An efficient cellular Potts model algorithm that forbids cell fragmentation. Comput Phys Commun 2016;208:54–63.

[btad773-B3] Graner F , Glazier JA. Simulation of biological cell sorting using a two-dimensional extended Potts model. Phys Rev Lett 1992;69:2013–6. 10046374 10.1103/PhysRevLett.69.2013

[btad773-B4] Hopcroft J , Tarjan R. Algorithm 447: efficient algorithms for graph manipulation. Commun ACM 1973;16:372–8.

[btad773-B5] Karypis G , Kumar V. A fast and high quality multilevel scheme for partitioning irregular graphs. SIAM J Sci Comput 1998;20:359–92.

[btad773-B6] Rackauckas C , Nie Q. DifferentialEquations.jl—a performant and feature-rich ecosystem for solving differential equations in Julia. JORS 2017;5:15.

[btad773-B7] Rens EG , Edelstein-Keshet L. From energy to cellular forces in the cellular Potts model: an algorithmic approach. PLoS Comput Biol 2019;15:e1007459. 31825952 10.1371/journal.pcbi.1007459 PMC6927661

[btad773-B8] Savill NJ , Hogeweg P. Modelling morphogenesis: from single cells to crawling slugs. J Theor Biol 1997;184:229–35. 31940735 10.1006/jtbi.1996.0237

[btad773-B9] Starruß J , de Back W, Brusch L et al Morpheus: a user-friendly modeling environment for multiscale and multicellular systems biology. Bioinformatics 2014;30:1331–2. 24443380 10.1093/bioinformatics/btt772 PMC3998129

[btad773-B10] Swat MH , Thomas GL, Belmonte JM et al Multi-scale modeling of tissues using CompuCell3D. Methods Cell Biol 2012;110:325–66. 22482955 10.1016/B978-0-12-388403-9.00013-8 PMC3612985

[btad773-B11] Szabó A , Merks RMH. Cellular Potts modeling of tumor growth, tumor invasion, and tumor evolution. Front Oncol 2013;3:87. 23596570 10.3389/fonc.2013.00087 PMC3627127

[btad773-B12] Wilson RJ. Introduction to Graph Theory. New York: Academic Press, 1972.

[btad773-B13] Wortel IMN , Niculescu I, Kolijn PM et al Local actin dynamics couple speed and persistence in a cellular Potts model of cell migration. Biophys J 2021;120:2609–22. 34022237 10.1016/j.bpj.2021.04.036 PMC8390880

